# Supplementary material for: The Systems Biology Research Tool: evolvable open-source software
Source: BMC Syst Biol. 2008 Jun 29;2:55. doi: 10.1186/1752-0509-2-55 (PMC2446383; doi:10.1186/1752-0509-2-55)
Supplement: Additional file 1 — SBRT Archive. An archive of the current version of the Systems Biology Research Tool. [file 1752-0509-2-55-S1.zip › sbrt-1.4.0/doc/developers_guide/api/sbrt/kernel/math/class-use/IntervalComparer.Diff.html]

Uses of Class sbrt.kernel.math.IntervalComparer.Diff


|  |  |  |
| --- | --- | --- |
| **Overview** \| **Package** \| **Class** \| **Use** \| **Tree** \| **Deprecated** \| **Index** \| **Help** | |  |
| PREV   NEXT | **FRAMES**    **NO FRAMES**     **All Classes** |  |


---


## **Uses of Class sbrt.kernel.math.IntervalComparer.Diff**

| Packages that use IntervalComparer.Diff | |
| --- | --- |
| **sbrt.kernel.math** | Provides classes and interfaces for creating objects relevant to mathematics. |
| **sbrt.kernel.utilities.proc** | Provides classes and interfaces for performing utility processes. |

Uses of IntervalComparer.Diff in sbrt.kernel.math

Methods in sbrt.kernel.math that return types with arguments of type IntervalComparer.Diff

`<V> java.util.Set<IntervalComparer.Diff<V>>`

`IntervalComparer.getDiffs(java.util.Map<? extends V,Interval> m1,
java.util.Map<? extends V,Interval> m2,
double tolerance)`

Uses of IntervalComparer.Diff in sbrt.kernel.utilities.proc

| Method parameters in sbrt.kernel.utilities.proc with type arguments of type IntervalComparer.Diff | |
| --- | --- |
| `void` | `IntervalComparison.run(ResultsManager<java.util.Set<IntervalComparer.Diff<V>>> resultsManager)`             Adds to the provided results manager the variables (keys) whose intervals (values) are unequal within the provided tolerance. |

---


|  |  |  |
| --- | --- | --- |
| **Overview** \| **Package** \| **Class** \| **Use** \| **Tree** \| **Deprecated** \| **Index** \| **Help** | |  |
| PREV   NEXT | **FRAMES**    **NO FRAMES**     **All Classes** |  |


---
